# Supplementary material for: Model-free estimation of COVID-19 transmission dynamics from a complete outbreak
Source: PLoS One. 2021 Mar 24;16(3):e0238800. doi: 10.1371/journal.pone.0238800 (PMC7990215; doi:10.1371/journal.pone.0238800)
Supplement: S1 Fig — The black line shows the age distribution of the New Zealand population (number of cases that would be expected if the cluster followed the same age distribution as the New Zealand population). The title of each panel describes the setting and location (Akl = Auckland; Chch = Christchurch; Qtn = Queenstown) associated with each cluster; n is the total number of recorded cases in the cluster. (DOCX) [file pone.0238800.s001.docx]

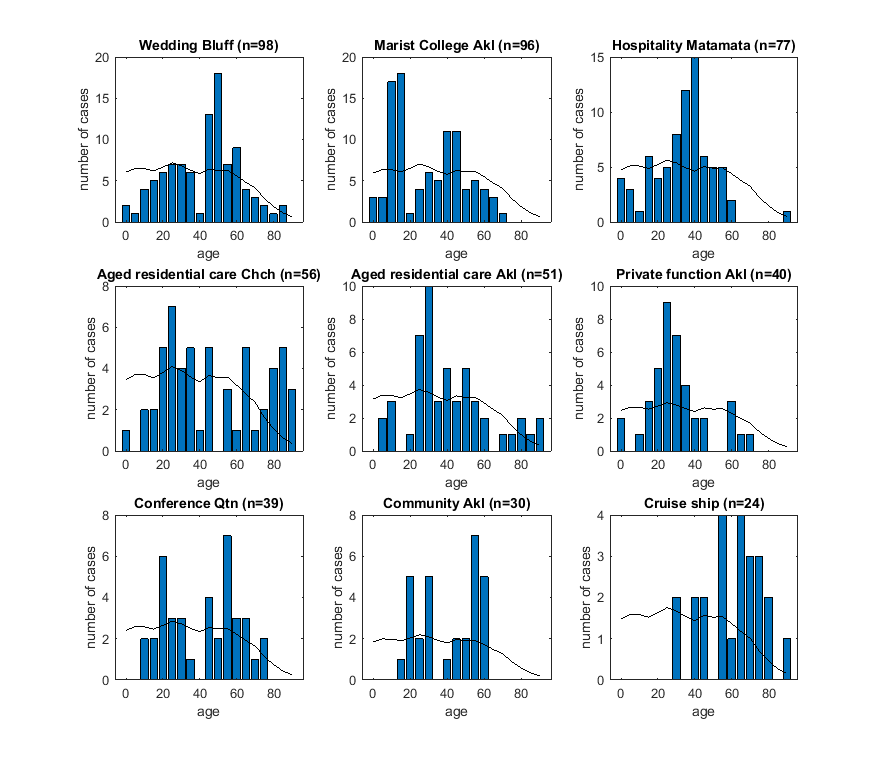


S1 Fig. Age distribution of cases in New Zealand’s nine largest clusters. The black line shows the age distribution of the New Zealand population (number of cases that would be expected if the cluster followed the same age distribution as the New Zealand population). The title of each panel describes the setting and location (Akl = Auckland; Chch = Christchurch; Qtn = Queenstown) associated with each cluster; n is the total number of recorded cases in the cluster.
